# Working from Home: Hybrid and Predominantly Home-Based Work in Relation to Work Environment, Job Satisfaction, and Health

**DOI:** 10.3390/ijerph23040524

**Published:** 2026-04-18

**Authors:** Michael Rosander

**Affiliations:** Department of Behavioural Sciences and Learning, Linköping University, 581 83 Linköping, Sweden; michael.rosander@liu.se

**Keywords:** working from home, hybrid work, remote work, role ambiguity, workload, job satisfaction, employee well-being, health, sleep

## Abstract

**Highlights:**

**Public health relevance—How does this work relate to a public health issue?**

- Working from home has become a widespread feature of modern work, raising questions about its implications for employees’ work environment and health.
- This study examines how different degrees of working from home—hybrid work and predominantly home-based work—relate to role ambiguity, job satisfaction, sleep problems, and general health.

**Public health significance—Why is this work of significance to public health?**

- The results show that hybrid work and predominantly home-based work are associated with different patterns of work environment and health outcomes.
- Poorer general health and more sleep problems were observed only among employees working predominantly from home.

**Public health implications—What are the key implications or messages for practitioners, policy makers and/or researchers in public health?**

- Patterns of outcomes differ across working-from-home arrangements rather than reflecting the mere presence of remote work.
- Organizations may need to balance the benefits of fewer interruptions with potential challenges related to role clarity, social interaction, and employee well-being.

**Abstract:**

Working from home has become an increasingly common feature of contemporary working life. The present study examined how different degrees of working from home relate to employees’ perceptions of the work environment, job satisfaction, and health. The analyses were based on a national probability sample of Swedish employees (N = 2331). A multivariate regression model was estimated to examine associations between three working arrangements—office, hybrid, and predominantly home-based work—and multiple employee outcomes. The results showed that hybrid work and predominantly home-based work were associated with different patterns of employee outcomes. Hybrid work was primarily related to higher role ambiguity and lower job satisfaction, whereas predominantly home-based work was associated with a broader set of outcomes, including poorer general health, more sleep problems, lower job satisfaction, higher role ambiguity, and lower perceived workload. Taken together, the findings suggest that patterns of associations differ across working-from-home arrangements rather than simply reflecting the mere presence of remote work. The study highlights the importance of distinguishing between different degrees of working from home when examining how remote work relates to employees’ work experiences and well-being.

## 1. Introduction

The organization of office work has undergone a substantial transformation in recent years. Although working from home (WFH) and other forms of telework existed well before 2020—typically on a limited or voluntary basis (e.g., [1])—the COVID-19 pandemic marked an unprecedented expansion in both their scale and intensity. During this period, large segments of the workforce shifted to home-based work, effectively establishing the feasibility of performing many office-based tasks outside the traditional workplace. In the years following the pandemic, WFH has persisted to varying degrees, with many employees continuing to work either part-time (hybrid) or predominantly from home. However, research has produced mixed findings regarding its implications for employees’ work environment, job attitudes, and health (e.g., [2,3]).

One reason for these mixed findings may lie in how WFH has been conceptualized and studied. The rapid normalization of WFH was paralleled by an equally rapid expansion of scholarly attention. Systematic and bibliometric reviews document a sharp increase in publications on remote and hybrid work after 2020, with a substantial proportion of empirical studies relying on data collected during the pandemic period [4,5]. As a result, observed associations may partly reflect broader psychosocial consequences of crisis conditions rather than stable effects of WFH arrangements per se. Moreover, reviews indicate considerable heterogeneity in how WFH has been operationalized. In many studies, WFH is treated as a dichotomous condition (remote vs. non-remote), and when intensity is examined, associations are frequently modeled as linear [3,5]. Such approaches implicitly assume proportional effects across increasing levels of WFH and may obscure potential differences between hybrid and predominantly home-based arrangements. In addition, existing research has often examined isolated outcome domains—such as well-being, job satisfaction, or work characteristics—rather than integrating work environment perceptions, job attitudes, and health outcomes within a unified analytical framework [5,6].

The present study addresses these limitations by examining how working from home—hybrid or predominantly home-based—is associated with employees’ perceptions of the work environment, job satisfaction, and health, compared to employees who do not work from home. Drawing on post-pandemic data collected in 2024 from a national probability sample and focusing on employees whose roles include office tasks, the study provides a population-level examination of WFH under what can be considered more typical working conditions, thereby offering insight into how different degrees of home-based work relate to multiple employee outcomes beyond crisis-driven contexts.

### 1.1. Conceptualizing Working from Home

Although telework and remote work have long been studied [1], these constructs have typically encompassed a broad range of arrangements performed away from the central workplace, including work at satellite offices, client sites, or other off-site locations. In contrast, working from home (WFH) refers specifically to performing one’s regular job tasks from the domestic environment. This distinction is theoretically important. The home context entails unique boundary dynamics between work and private life, altered patterns of social interaction, and potential challenges related to role clarity and informal coordination that may not be captured in earlier telework research.

In the present study, WFH is defined as performing regular work tasks from home at least some of the time during a typical workweek. Employees working from home up to two days per week are classified as engaging in *hybrid work*, whereas those working from home three or more days per week are classified as engaging in predominantly home-based work (*majority WFH*, i.e., working from home the majority of the workweek). To ensure meaningful comparisons, analyses are restricted to employees whose work includes office tasks. By excluding respondents who do not engage in office work, the study reduces the risk that observed differences reflect structural constraints associated with occupations that require physical presence, such as manual or frontline service work.

A further challenge in WFH research concerns selection processes. Access to and use of WFH are unevenly distributed across occupational groups and are more common among highly educated employees, knowledge workers, managers, and those with higher income and greater decision latitude [2,5,7]. Such patterns have been consistently observed both prior to and following the COVID-19 pandemic [5,7]. Consequently, differences between employees who work from home and those who do not may partly reflect compositional variation in socioeconomic position and job design rather than the work arrangement itself. Previous research has highlighted the importance of accounting for structural, demographic, and work design factors when examining associations between WFH and employee outcomes [2,7]. In particular, job control represents a theoretically central potential confound. Higher decision latitude is consistently associated with more favorable work environment perceptions, job satisfaction, and well-being [8,9], and is also more prevalent in occupations where WFH is feasible [5,7]. Failing to account for such factors risks attributing resource-related advantages to WFH per se rather than to underlying characteristics. To reduce the risk of confounding, the analyses in the present study adjust for managerial position, gender, sector, education, age, income, and job control.

### 1.2. Working from Home and the Work Environment

From a work design perspective [10], working from home represents a substantial change in the structural and social conditions under which work is performed. Changes in where and how work is carried out may alter both the demands and available resources, which in turn shape employees’ perceptions of their work environment [8]. Traditional office environments facilitate coordination, feedback, and role clarification through frequent face-to-face interactions and informal exchanges among colleagues and supervisors. When work is relocated to the home environment, these interaction patterns are partly replaced by digitally mediated communication, which may alter how information is exchanged, how expectations are communicated, and how tasks are coordinated [7,11]. Reviews of telework and remote work research suggest that such changes in communication and coordination processes constitute a central pathway through which remote work arrangements influence employees’ perceptions of their work environment [2,5].

One work environment characteristic that may be particularly sensitive to these changes is *role ambiguity*, defined as uncertainty regarding expectations, responsibilities, and performance criteria associated with one’s role [12]. According to role theory, organizations function as systems of roles defined by expectations communicated to role occupants [13]. Role clarity therefore depends on the availability of information and ongoing interaction through which expectations can be communicated and negotiated [12]. When work is performed primarily from home, opportunities for spontaneous clarification and informal feedback may be reduced, potentially increasing uncertainty about priorities and expectations. Although digital communication can compensate for some of these challenges, it may also reduce the immediacy and richness of interaction compared to in-person exchanges [11]. At the same time, remote work arrangements may increase autonomy and discretion in task execution, which could allow employees greater flexibility in structuring their work and thereby reduce ambiguity [7,14]. Consequently, theoretical expectations regarding the relationship between working from home and role ambiguity remain mixed.

A second work environment dimension that may be influenced by working from home is *workload*, reflecting the perceived intensity and demands associated with work tasks. From the perspective of the job demands–resources model [8], work arrangements may simultaneously alter both job demands and job resources. Working from home may reduce certain demands, such as commuting and exposure to workplace interruptions, while increasing others, including digital communication demands, coordination efforts, and blurred boundaries between work and non-work activities [2,11]. Research suggests that hybrid work arrangements may improve some aspects of work organization—such as control over workflow and exposure to environmental distractions—while more extensive remote work may increase perceived work intensity or time pressure [5,14]. As a result, mechanisms pointing in both directions are likely to be at play, meaning that the overall relationship between working from home and workload is theoretically ambiguous.

Importantly, the extent to which employees work from home may shape these dynamics. Hybrid arrangements allow employees to combine periods of remote work with continued presence in the workplace, which may facilitate coordination, informal interaction, and ongoing role clarification. In contrast, predominantly home-based work involves fewer opportunities for face-to-face interaction and informal communication. Distinguishing between hybrid and predominantly home-based work may therefore help clarify how working from home relates to work environment perceptions. Because perceptions of role clarity and workload constitute central aspects of employees’ psychosocial work environment, differences in these conditions may also have implications for employees’ job attitudes and well-being.

### 1.3. Working from Home and Job Satisfaction

Job satisfaction represents one of the most frequently examined attitudinal outcomes in research on telework and remote work [5,7]. From the perspective of the job demands–resources model [8], working conditions characterized by greater resources and manageable demands are generally associated with more positive job attitudes. Working from home may influence both sides of this balance. On the one hand, remote work arrangements may increase job resources by providing greater autonomy, flexibility, and control over work schedules and work environments [3,7]. On the other hand, reduced social interaction, coordination challenges, and potential difficulties in maintaining clear boundaries between work and private life may represent additional demands that can undermine positive job attitudes [2,11].

Findings regarding the relationship between working from home and job satisfaction are therefore mixed. Meta-analytic evidence generally suggests modest positive associations between telework and job satisfaction, although effect sizes tend to be small and vary depending on contextual conditions [3,7]. At the same time, some studies indicate that the extent of remote work may matter, with moderate levels of remote work sometimes associated with more favorable outcomes than either no remote work or extensive remote work [15,16]. Such findings suggest that treating working from home as a simple dichotomous condition may obscure meaningful differences in how various degrees of remote work relate to employees’ job attitudes.

One possible interpretation is that different degrees of working from home involve distinct combinations of job demands and resources. Hybrid arrangements may allow employees to benefit from increased flexibility and autonomy while maintaining access to face-to-face interaction, informal coordination, and organizational support available in the workplace. In contrast, predominantly home-based work may involve reduced opportunities for social interaction and informal feedback, which could attenuate some of the potential benefits of remote work. Distinguishing between hybrid and predominantly home-based work may therefore help clarify how working from home relates to employees’ job satisfaction.

Because job satisfaction represents a central indicator of employees’ overall work-related well-being, examining how different degrees of working from home relate to job satisfaction may also provide insight into broader implications for employee health and well-being.

### 1.4. Working from Home and Health

Beyond job attitudes, an important question concerns whether working from home is associated with employees’ health outcomes. Reviews of telework and remote work research suggest that remote work arrangements may influence a range of health-related indicators, including psychological well-being, stress, and work–life balance [2,6]. From the perspective of occupational health research, such associations are often understood in terms of how work arrangements shape the balance between job demands and resources, which in turn may influence employees’ mental and physical health [8].

Evidence on the health implications of working from home remains mixed. Some studies have reported beneficial effects, such as reduced stress and improved work–life balance, whereas others have identified potential risks related to social isolation, blurred work–home boundaries, and increased work intensity [2,11]. Importantly, a substantial proportion of empirical studies on remote work and health rely on data collected during the COVID-19 pandemic [5]. Because this period was characterized by widespread uncertainty, health concerns, and disruptions to everyday life, observed associations between working from home and health outcomes may partly reflect broader psychosocial consequences of crisis conditions rather than stable effects of remote work arrangements.

Recent studies have begun to examine sleep as a potentially important outcome in the context of working from home [17,18]. As a health-related outcome, sleep may reflect the cumulative effects of work environment conditions and job-related experiences associated with different working arrangements. Working from home may influence sleep through several pathways. The absence of commuting and greater flexibility in work schedules may allow employees to align work and sleep patterns more closely with individual preferences. At the same time, increased digital communication, extended working hours, and blurred boundaries between work and private life may negatively affect sleep quality and recovery [11,18]. Empirical findings regarding sleep and related health indicators remain inconsistent [2].

As with job satisfaction, the extent to which employees work from home may play an important role. Hybrid arrangements may provide flexibility and autonomy while still maintaining opportunities for social interaction and organizational support available in the workplace. In contrast, predominantly home-based work may involve fewer opportunities for face-to-face interaction and potentially greater challenges related to boundary management and social connectedness. Examining different degrees of working from home may therefore contribute to a more nuanced understanding of how remote work arrangements relate to employees’ health and sleep.

### 1.5. The Present Study

Taken together, previous research suggests that working from home may influence employees’ work environment perceptions, job attitudes, and health, but evidence regarding different degrees of home-based work remains limited. The present study therefore examines how hybrid and predominantly home-based working arrangements relate to employees’ perceptions of the work environment, job satisfaction, and health. Using data collected in 2024 from a national probability sample of employees whose work includes office tasks, the study provides an opportunity to examine working from home in a post-pandemic context with more typical remote work arrangements.

*Research Question 1.* How do employees who do not work from home, engage in hybrid work, or engage in predominantly home-based work differ in demographic and job-related characteristics?

*Research Question 2.* How are different degrees of working from home associated with employees’ perceptions of the work environment (role ambiguity and workload), job satisfaction, and health (general health and sleep problems)?

The present study contributes to the literature by distinguishing between hybrid and predominantly home-based work, addressing a limitation in previous research that often treats working from home as a dichotomous condition. By examining multiple work environments, attitudinal, and health outcomes within a multivariate framework, the study also provides a more integrated understanding of how different working-from-home arrangements relate to employees’ experiences, and offers a basis for a more differentiated account of how working from home relates to job demands and resources.

## 2. Materials and Methods

The study draws on data from a national probability sample of the Swedish working population aged 18 years and older and employed in workplaces with at least ten employees. This criterion excludes very small workplaces, which may differ in organizational structure and are subject to different regulatory requirements in the Swedish context. Sampling and data collection were administered by Statistics Sweden (https://www.scb.se/en/, accessed on 16 April 2026), the national statistical agency. Data were collected between September and October 2024 (response rate 25%). Available information provided by Statistics Sweden indicates that respondents were somewhat more likely to be women, older, and to have higher education and income compared to the working population. However, this information is not available specifically for employees whose work includes office tasks. Information about the study and all materials required for informed consent were distributed digitally to individuals with an active digital mailbox and by post to those without. Up to three reminders were issued to non-respondents, with the second reminder sent by post to all who had not yet responded. Following the completion of data collection, Statistics Sweden supplemented the dataset with register-based information from the Swedish National Population Register (e.g., sex, age, income, and educational attainment). The dataset was anonymized before access was granted to the researchers. A total of 3307 respondents participated in the survey. For the purposes of the present study, analyses were restricted to respondents who reported that their work includes office tasks. Respondents who indicated that they do not engage in office work were excluded in order to reduce the risk that comparisons reflect structural constraints associated with occupations that inherently require physical presence. The study was conducted in accordance with the principles of the Declaration of Helsinki and was approved by the Swedish Ethical Review Authority (Protocol No. 2023-06603-01).

### 2.1. Participants

The final analytic sample consisted of 2331 respondents whose work includes office tasks. Of the participants, 55% were women and 45% men, and the mean age was 48.22 years (SD = 10.82). On average, participants had worked 10.57 years (SD = 9.53) at their current workplace. The majority held a permanent position (96%), and 21% reported having a managerial role. With regard to educational attainment, 12% had completed compulsory education, 32% upper secondary education, and 56% held a university degree.

### 2.2. Measures

*Working from home* was assessed using two survey items. Respondents were first asked whether they worked from home at least some of the time (“Do you work from home at least part of the time?”, yes/no). Those who responded affirmatively were asked how many days per week they had, on average, worked from home during the past month. Response options ranged from less than one day to five days per week. Based on these responses, participants were categorized into three groups: employees who did not work from home, employees engaging in hybrid work (up to two days per week), and employees engaging in predominantly home-based work (three or more days per week).

*Role ambiguity* was assessed using three items from the role ambiguity scale developed by Rizzo and colleagues [12], capturing ambiguity regarding roles, responsibilities, and task boundaries at the workplace. An example item is “My role, responsibilities, and tasks at the workplace are very unclear and ambiguous”. Responses were given on a seven-point Likert scale ranging from not at all to completely. Internal consistency was satisfactory (Cronbach’s α = 0.80).

*Workload* was measured using four items from the effort scale of the effort–reward imbalance model by Siegrist and colleagues [19], capturing perceived time pressure, difficulties keeping up with work tasks, the need to work overtime, and interruptions that affect task completion. An example item is “I find it difficult to keep up with all my work tasks”. Responses were recorded on a seven-point Likert scale ranging from not at all to completely. Internal consistency was good (Cronbach’s α = 0.83).

*Job satisfaction* was measured using four items reflecting affective and evaluative aspects of employees’ satisfaction with their current job [20]. An example item is “I like my current job”. Responses were given on a seven-point Likert scale ranging from not at all to completely. The scale showed high internal consistency (Cronbach’s α = 0.90).

*General health* was assessed using a single item asking respondents to rate their overall health during the past 12 months (“In general, how would you rate your health over the past 12 months?”). Responses were provided on a five-point scale ranging from very poor to very good. Single-item measures of general health are widely used in survey research and have been shown to be reliable and valid indicators of overall health [21].

*Sleep problems* were measured using three items capturing distress related to difficulties initiating sleep, maintaining sleep, and waking up too early, which represent core aspects of sleep disturbance [22]. Participants indicated the degree of distress associated with each problem on a four-point scale ranging from no distress to severe distress. Internal consistency for the scale was acceptable (Cronbach’s α = 0.74).

### 2.3. Control Variables

To reduce the risk of confounding, several demographic and job-related variables were included as controls. Access to and use of working-from-home arrangements are unevenly distributed across occupational and demographic groups [5,7], meaning that employees who work from home may differ systematically from those who do not in terms of job characteristics, socioeconomic position, and work-related resources. Such differences may also be associated with employees’ perceptions of the work environment, job satisfaction, and health. The analyses controlled for managerial position, sex, sector, educational attainment, age, income, and perceived job control.

Managerial position was assessed with a direct survey question asking whether the respondent held a managerial role. Register-based information from the Swedish National Population Register was used to obtain data on registered sex, age, educational attainment, sector (public vs. private), and income.

Perceived *job control* was measured using three items reflecting employees’ influence over work tasks and decisions [20]. An example item is “I have a great deal of influence over the amount of work I am given”. Responses were recorded on a seven-point Likert scale ranging from not at all to completely. Internal consistency was acceptable (Cronbach’s α = 0.74).

### 2.4. Sensitivity Analysis

As a sensitivity analysis, the main model was re-estimated including psychological distress as an additional control variable. Psychological distress may influence how employees perceive their work environment, their level of job satisfaction, and their self-reported health [23], and may also be related to the extent to which employees work from home. Including this variable allowed examination of whether the associations between working from home and the study outcomes remained when accounting for potential confounding related to underlying psychological distress.

Psychological distress was measured using the Hospital Anxiety and Depression Scale [24], a 14-item instrument assessing symptoms of anxiety and depression during the past week on a four-point scale ranging from 0 (not at all) to 3 (most of the time). Example items include “I feel tense or ‘wound up’” and “I have lost interest in my appearance”. Internal consistency for the scale was high (Cronbach’s α = 0.91).

In addition, to assess the robustness and generalizability of the descriptive findings, the demographic and job-related characteristics were re-estimated using survey-weighted analyses based on calibrated population weights provided by Statistics Sweden.

All results are presented in the Supplemental Materials (Tables S1 and S2).

### 2.5. Data Analysis

Data analyses were conducted using Stata 19.5. Descriptive statistics and bivariate correlations were first examined for all study variables. To address Research Question 1, differences in demographic and job-related characteristics across the three working arrangements (not working from home, hybrid work, and predominantly home-based work) were examined using descriptive statistics and group comparisons. Group differences were tested using chi-square tests for categorical variables and one-way analyses of variance (ANOVA) for continuous variables. For continuous variables, Bonferroni-adjusted pairwise comparisons were used to identify group differences, whereas for categorical variables, post hoc pairwise comparisons of proportions (z-tests) were conducted within each WFH category, with Bonferroni adjustment.

To address Research Question 2, a multivariate regression analysis was conducted to examine how different degrees of working from home were associated with employees’ perceptions of the work environment (role ambiguity and workload), job satisfaction, and health outcomes (general health and sleep problems). Working from home was entered as a categorical variable distinguishing between employees who did not work from home, those engaging in hybrid work, and those engaging in predominantly home-based work. The model adjusted for managerial position, sex, sector (public vs. private), educational attainment, age, income, and perceived job control. Because several outcomes were conceptually related, the equations were estimated simultaneously using multivariate regression, allowing the associations between working from home and multiple outcomes to be examined within a unified analytical framework. Following model estimation, multivariate F-tests were conducted to evaluate the joint significance of the working-from-home coefficients across all outcomes, as well as the joint effects of hybrid work and predominantly home-based work separately. Additional tests compared the coefficients for hybrid work and predominantly home-based work across the set of outcomes and for each outcome individually. A statistically significant test indicates that the coefficients differ across working-from-home categories, either across outcomes or within a given outcome.

Income was obtained from register-based data from the Swedish National Population Register. Because the distribution was positively skewed, extreme values were limited by winsorizing the variable at the 1st and 99th percentiles, after which the variable was log-transformed for use in the analyses. These transformations were applied to reduce the influence of extreme values and improve the distributional properties of the variable for regression analysis.

All statistical tests were two-tailed and evaluated at a significance level of *p* < 0.05. The distributions of the study variables and model residuals were inspected, and residual plots were examined to assess model assumptions. While minor deviations from normality were observed, no substantial concerns were indicated with respect to linearity or homoscedasticity. Given the large sample size, such deviations were not considered to meaningfully affect the estimates.

## 3. Results

Descriptive statistics and correlations among the study variables are presented in Table 1. On average, employees reported relatively good general health and low to moderate levels of sleep problems. The correlations were generally in the expected directions and ranged from small to moderate in magnitude. Better health and fewer sleep problems were associated with higher job satisfaction and job control, and with lower role ambiguity and workload. Psychological distress showed moderate to strong associations with all outcome variables. Notably, job satisfaction, role ambiguity, job control, and psychological distress were relatively strongly interrelated, indicating a consistent pattern across key aspects of the work environment and well-being.

To address Research Question 1, demographic and job-related characteristics were compared across employees who never worked from home, engaged in hybrid work, or engaged in predominantly home-based work (majority WFH). The results are presented in Table 2. With regard to demographic characteristics, employees who worked from home were, overall, somewhat younger than those who never worked from home. No significant differences were observed for sex. Clear differences emerged for education. Among employees with compulsory education, a majority reported never working from home (58%), compared with those with upper secondary (43%) and university education (33%). In contrast, employees with a university degree were more likely to engage in hybrid work (48%) than those with upper secondary (40%) or compulsory education (29%). Employees with young children were less likely to report never working from home (29% vs. 41%) and more likely to engage in hybrid work (51% vs. 42%) compared with those without young children, whereas no significant differences were observed for predominantly home-based work. Differences were also evident in socioeconomic and job-related characteristics. Employees working from home reported higher income levels and higher levels of job control than those who never worked from home, with no differences between hybrid and majority WFH. Employees who never worked from home also reported longer tenure than those working from home. With regard to sector, employees in the public sector were more likely to report never working from home (42% vs. 36%), whereas employees in the private sector were more likely to engage in predominantly home-based work (21% vs. 15%). Finally, among employees in managerial positions, a larger proportion engaged in hybrid work (49%) compared with non-managerial employees (41%), whereas no significant differences were observed for predominantly home-based work. Although several group differences were statistically significant, the proportion of explained variance for continuous variables ranged from 0.006 to 0.027 (η^2^), and associations for categorical variables ranged from 0.03 to 0.12 (Cramér’s V).

To address Research Question 2, a multivariate regression model was estimated to examine how hybrid work and predominantly home-based work were associated with employees’ perceptions of the work environment (role ambiguity and workload), job satisfaction, and health outcomes (general health and sleep problems), controlling for demographic and job-related characteristics. The results are presented in Table 3. A multivariate test indicated that working from home was significantly associated with the combined set of outcomes, *F*(10, 2204) = 7.26, *p* < 0.001. When examined separately, both hybrid work, *F*(5, 2204) = 6.64, *p* < 0.001, and predominantly home-based work, *F*(5, 2204) = 10.49, *p* < 0.001, were significantly related to the outcomes. The coefficients also differed significantly between hybrid work and predominantly home-based work, *F*(5, 2204) = 5.31, *p* < 0.001. Overall, a clear pattern emerges in which predominantly home-based work is associated with a broader range of outcomes, whereas hybrid work shows more selective associations.

Examining the outcome variables separately, hybrid work was not significantly associated with general health, sleep problems, or workload. However, employees engaging in hybrid work reported lower job satisfaction, *b* = −0.15, *p* = 0.001, and higher role ambiguity, *b* = 0.26, *p* < 0.001, compared with employees who never worked from home. Predominantly home-based work (majority WFH) showed a broader pattern of associations. Compared with employees who never worked from home, those engaging in majority WFH reported poorer general health, *b* = −0.21, *p* < 0.001, more sleep problems, *b* = 0.10, *p* = 0.009, lower job satisfaction, *b* = −0.24, *p* < 0.001, higher role ambiguity, *b* = 0.31, *p* < 0.001, and lower workload, *b* = −0.18, *p* = 0.004. Notably, whereas the other outcomes were less favorable, workload showed an opposite pattern. Direct comparisons between hybrid work and predominantly home-based work further showed that majority WFH was associated with poorer health, more sleep problems, and lower workload than hybrid work (all *p* < 0.05). No significant differences were observed between the two groups with regard to job satisfaction or role ambiguity. In terms of magnitude, predominantly home-based work was most strongly related to role ambiguity (β = 0.29), followed by job satisfaction and general health (β = −0.22) and sleep problems and workload (β = 0.16). For hybrid work, the corresponding coefficients were β = 0.25 for role ambiguity and β = −0.14 for job satisfaction. More specifically, predominantly home-based work was associated with poorer health, more sleep problems, and lower workload compared with hybrid work, whereas no differences were observed for role ambiguity or job satisfaction.

As sensitivity analyses, the model was re-estimated including psychological distress as an additional control variable, and all descriptive comparisons in Table 2 were re-estimated using survey-weighted analyses based on calibrated population weights. The overall pattern of results remained unchanged, with no substantive changes in the direction or significance of the findings (see Supplemental Materials, Tables S1 and S2).

## 4. Discussion

The present study examined how different degrees of working from home relate to employees’ perceptions of the work environment, job satisfaction, and health. Overall, the findings indicate that hybrid work and predominantly home-based work are associated with distinct patterns of employee outcomes compared with not working from home, even after accounting for demographic and job-related factors linked to both working from home and more favourable work conditions. More specifically, hybrid work was primarily associated with higher role ambiguity and lower job satisfaction, whereas predominantly home-based work was related to a broader range of outcomes, including these as well as poorer general health and more sleep problems, but also lower perceived workload. This overall pattern remained stable in a sensitivity analysis adjusting for psychological distress. The findings also point to systematic differences in demographic and job-related characteristics across working-from-home arrangements. Employees who worked from home, regardless of the extent, tended to be younger, more highly educated, and more likely to have young children. They also reported shorter tenure but higher levels of job control and income. Predominantly home-based work was more common in the private sector, whereas managerial roles were most prevalent among hybrid workers. These patterns also remained essentially unchanged when applying calibrated population weights to account for potential non-response bias.

An important implication of these findings concerns the degree to which employees work from home. Much of the existing research has treated working from home as a relatively uniform condition, often contrasting employees who work remotely with those who do not (e.g., [1,5,7]). The present findings suggest that such dichotomous distinctions may obscure meaningful differences between forms of remote work. In particular, hybrid work and predominantly home-based work were associated with different patterns of employee outcomes. This pattern is consistent with arguments in the work design literature that relatively small changes in where work is performed may alter important features of the work context, including access to information, opportunities for coordination, and patterns of social interaction [10]. When employees work from home only part of the week, they may remain embedded in the everyday coordination processes of the workplace, whereas predominantly home-based work may involve a more substantial shift in how work is organized and experienced. The findings therefore highlight the importance of distinguishing between different working-from-home arrangements rather than treating remote work as a uniform phenomenon.

### 4.1. The Work Environment

With regard to perceptions of the work environment, the findings suggest that even limited forms of working from home may be linked to changes in everyday coordination processes, as both hybrid and predominantly home-based work were associated with higher levels of role ambiguity. According to role theory, expectations regarding responsibilities and performance are not only formally defined but also continuously communicated and negotiated through everyday interaction within organizations [13]. When employees work from home, opportunities for such informal feedback and spontaneous coordination may become more limited. Communication tends to be more structured and scheduled, while opportunities for informal exchanges and quick clarifications are reduced, which may make it more difficult to access and share relevant information [25]. The fact that higher role ambiguity was already evident among employees engaged in hybrid work may partly reflect what can be described as coordination asymmetry, in that even when employees are present in the office part of a typical workweek, other key actors are not necessarily present at the same time. When collaboration takes place across spatially dispersed settings, shared context may weaken, and misunderstandings and coordination problems become more likely [26]. In such situations, employees may have fewer opportunities to clarify expectations regarding their roles through ongoing interaction. At the same time, employees working from home reported higher levels of job control, which could in principle reduce reliance on clearly defined role expectations in performing one’s own tasks. However, from a role-theoretical perspective, ambiguity emerges within a system of interdependent expectations [13,27]. Role clarity therefore depends not only on understanding one’s own responsibilities but also on having clear expectations regarding the actions of others. Consequently, even when employees experience greater autonomy over their own work, coordination processes may still become less predictable when work is performed partly from home.

A somewhat different pattern emerged for perceived workload. Employees working predominantly from home reported a lower workload than those who did not work from home, whereas no such difference was observed for hybrid work. One possible explanation is that working from home may reduce exposure to the interruptions and spontaneous requests that often arise in office environments. Frequent interruptions can fragment ongoing work and require employees to repeatedly shift attention between tasks, thereby increasing the perceived intensity of work [28]. When employees work remotely, opportunities for informal interaction and ad hoc coordination are typically lower, which may allow work to proceed in longer, more continuous periods and thereby reduce perceived workload [25].

Taken together, these findings suggest that the same processes that may reduce workload may also contribute to lower role clarity. Informal interactions in the workplace often serve a dual function: while they can generate interruptions and spontaneous requests that increase workload, they also provide opportunities to clarify expectations regarding tasks, responsibilities, and performance [13]. When such interactions become less frequent in remote work settings, employees may experience fewer interruptions but also fewer opportunities to negotiate and reaffirm role expectations. This may help explain why reduced workload and increased role ambiguity were observed simultaneously.

From a job demands–resources perspective [8], these findings suggest that working from home may involve simultaneous changes in different types of job demands rather than a uniform increase or decrease in overall demands. While certain demands, such as interruptions and time pressure, may be reduced, other demands related to coordination and role clarity may increase. This may help explain why predominantly home-based work was associated with lower workload but higher role ambiguity, alongside less favorable outcomes in terms of job satisfaction and health. In this sense, the findings point to a more differentiated understanding of remote work, where changes in the composition of demands and resources, rather than their overall level, may be central for understanding employee outcomes.

### 4.2. Job Satisfaction

Previous research on telework has produced mixed findings regarding job satisfaction. However, several studies and reviews have reported positive associations [1,7], and recent research on hybrid work likewise suggests that remote work does not necessarily reduce employee satisfaction [29]. In the present study, employees working from home reported lower job satisfaction, both among those engaged in hybrid work and those working predominantly from home. Importantly, the analyses accounted for factors that both increase the likelihood of working from home and are commonly associated with higher job satisfaction, such as job control, income, education, and managerial position [8,30].

The lower levels of job satisfaction observed among employees working from home may partly reflect the social functions that work serves. Employment not only provides income but also opportunities for regular social contact and shared experiences with others [31]. From a job demands–resources perspective, social interaction and support at work constitute key job resources that contribute to motivation and positive work attitudes [8]. When work is performed from home, however, opportunities for informal interaction and spontaneous exchanges may become more limited. Research on remote work suggests that communication in distributed settings tends to become more structured and task-focused [25], potentially reducing the social aspects of everyday work life. Social relationships at work play an important role in employee well-being and job attitudes [32], and fewer opportunities for such interaction may therefore help explain the lower job satisfaction among employees working from home. In addition, other mechanisms may contribute to this pattern. Although the analyses accounted for several key factors related to both working from home and job satisfaction, unobserved differences between employees may still play a role. For example, employees who work from home may differ systematically from those who do not, reflecting residual self-selection processes. At the same time, working from home may be associated with differences in job content and work organization, such as more independent and less socially embedded tasks, as well as higher expectations of availability, challenges in managing boundaries between work and private life, and reduced visibility within the organization. These factors may, in turn, have implications for career development, recognition, and overall job satisfaction.

### 4.3. Health

With regard to health outcomes, employees working predominantly from home reported poorer general health and more sleep problems than those who did not work from home, whereas no such differences were observed for hybrid work. Although previous research has reported varying associations between remote work and employee well-being [7], emerging evidence suggests that its consequences may depend on the extent to which work is performed from home rather than on the mere presence of remote work. A few studies, for example, report curvilinear relationships in which moderate levels of remote work are associated with more favorable outcomes than either very low or very high levels [16,33,34].

One possible interpretation is that hybrid work allows employees to combine some of the advantages of working from home with continued access to important workplace resources. From a job demands–resources perspective, employee health and well-being depend on the balance between job demands and the availability of job resources that support coping and recovery [8]. Working from home may influence this balance by altering employees’ access to resources embedded in the workplace, such as informal feedback, opportunities for coordination, and everyday social interaction with colleagues. When employees work from home only part of the week, they may still maintain regular access to these resources through their presence at the workplace. Predominantly home-based work, in contrast, may represent a more substantial shift in everyday work arrangements, potentially reducing access to such resources, which may in turn be related to recovery and well-being.

A related explanation concerns the work environment patterns identified in the present study. Employees working from home reported higher levels of role ambiguity, suggesting that coordination processes and expectations regarding responsibilities may become less clear when work is performed partly or largely from home. Unclear expectations and coordination challenges can create cognitive strain and make it more difficult for employees to disengage from work during non-work hours [13,35], which may, in turn, be relevant for understanding differences in recovery and sleep. Although the cross-sectional design does not allow conclusions about causal mechanisms, future longitudinal research could examine whether role-related stressors associated with extensive remote work contribute to sleep disturbances and longer-term health outcomes.

Another possible explanation concerns boundaries between work and non-work domains. When work is performed primarily from home, the spatial and temporal boundaries separating work from private life may become less clearly defined. Research on recovery from work suggests that difficulties psychologically detaching from work during leisure time can interfere with sleep and recovery [35]. Such boundary dynamics may become particularly relevant when working from home constitutes the predominant mode of work rather than an occasional or hybrid arrangement.

Finally, it is also possible that the reduced opportunities for social interaction that accompany extensive remote work play a role. Work fulfills important social functions in everyday life, including providing a context for regular interaction and shared experiences with others [31]. When work is performed largely from home, opportunities for such interaction may become more limited, which could influence aspects of well-being over time.

Taken together, these explanations suggest that the differences observed in the present study may be understood in terms of how varying degrees of working from home shape employees’ access to key workplace resources and everyday work processes. Hybrid work may allow employees to retain access to such resources while benefiting from the flexibility associated with remote work, whereas predominantly home-based work may involve greater changes in coordination processes, social interaction, and boundary management, which may in turn affect recovery and health.

### 4.4. Demographic and Job-Related Differences

The observed differences in who works from home highlight that access to remote work is linked to structural and job-related resources. Employees who work from home tend to occupy positions characterized by greater autonomy, higher income, and greater job control [1,7]. Differences between the two working-from-home arrangements were generally modest, and employees across groups were broadly similar across many of the characteristics examined. In this sense, the opportunity to work from home may partly function as a privilege associated with resource-rich jobs that offer greater flexibility in how and where work is performed. At the same time, several of these factors are also related to more favorable work experiences and well-being. Taking such differences into account is therefore important when examining outcomes associated with remote work, as more favorable results may otherwise reflect underlying differences in job resources and working conditions rather than working from home by itself, even when accounting for a range of observed factors.

The findings regarding managerial positions are noteworthy. Managers were more likely than non-managerial employees to engage in hybrid work, and for predominantly home-based work, the proportions were about the same in both groups. This means that a non-negligible proportion of managers reported working predominantly from home. Leadership roles typically involve coordination, support, and ongoing interaction with employees, and research on destructive leadership has shown that passive or absent leadership can have negative consequences for employees and the work environment [36,37]. Although working from home does not necessarily imply reduced leadership engagement, extensive remote work among managers may reduce opportunities for everyday interaction and visibility, which may increase the risk that leadership becomes more distant or less accessible to employees. Managers’ work arrangements may also influence broader workplace norms. Leaders often function as role models whose behavior signals what is considered appropriate or expected within organizations [38]. Consequently, managers’ use of remote work may shape employees’ perceptions of expectations regarding presence, availability, and ways of organizing work. In this sense, managers’ decisions about working from home may have implications beyond their own working conditions and influence how remote work practices develop within organizations.

### 4.5. Practical Implications

The present findings highlight that how work is experienced differs across working-from-home arrangements, as hybrid work and predominantly home-based work were associated with different patterns of outcomes. Working from home involves a trade-off between different aspects of the work environment. On the one hand, it may involve fewer interruptions and spontaneous requests that often arise in office settings, allowing employees to work in more focused and uninterrupted periods. On the other hand, the results suggest that reduced opportunities for informal interaction may make it more difficult to clarify expectations regarding roles and responsibilities, and reduced social interaction may also negatively affect aspects of health and well-being. Taken together, this suggests that working from home may shift the composition of job demands rather than simply increasing or decreasing them overall, and that the optimal balance between these aspects is likely to vary across occupations, tasks, and organizational contexts.

The results also highlight the importance of organizations establishing clearer communication routines, expectations regarding responsibilities, and opportunities for regular coordination when employees work from home. Particular attention may also be warranted for managerial roles. A majority of managers in the present study worked from home at least part of a typical week (64%), although most engaged in hybrid work (49%). Because leadership roles often rely on visibility, coordination, and everyday interaction with employees, extensive remote work among managers may reduce opportunities for leadership presence and accessibility. Organizations may therefore need to consider how managerial work arrangements may shape not only managers’ own working conditions but also coordination processes and expectations within the broader work environment.

### 4.6. Strengths and Limitations

The present study has several strengths that should be noted. First, the analyses were based on a national probability sample of employees, which enhances the generalizability of the findings (employees whose work includes office tasks). Second, the relatively large sample size allowed the simultaneous examination of multiple outcomes related to the work environment, job satisfaction, and health. Estimating these outcomes within a multivariate framework made it possible to assess how different degrees of working from home were associated with several aspects of employees’ work experiences in a unified analytical model. Third, the analyses accounted for a range of demographic and job-related factors that are both associated with the likelihood of working from home and known to be related to more favorable work conditions, including job control, income, education, sector, and managerial position [8,30]. Finally, the study distinguishes between hybrid work and predominantly home-based work, allowing a more nuanced examination of remote work than studies that treat working from home as a dichotomous condition.

At the same time, several limitations should be acknowledged. First, the cross-sectional design limits conclusions about causal relationships and directionality. Although the analyses adjusted for a number of relevant factors, the possibility of residual confounding and selection effects cannot be ruled out. In addition, some variables included as covariates (e.g., job control and psychological distress) may also represent underlying mechanisms, and adjusting for them may therefore result in more conservative estimates of the associations. Furthermore, reverse causality cannot be excluded, as employees with poorer health or lower job satisfaction may be more likely to work from home. Future longitudinal research is needed to examine how changes in work-from-home arrangements relate to changes in work experiences and health over time. Second, the measures were based on self-reports, which may introduce common method bias [39]. However, the inclusion of multiple outcomes and the focus on different aspects of work experiences reduce concerns that the findings reflect a single underlying response tendency. In addition, general health was assessed using a single item with a 12-month recall period, which may introduce recall bias and limit temporal alignment with current working arrangements, and does not capture specific dimensions of health, which may limit the level of detail in the assessment. The measure of sleep problems reflects distress associated with sleep difficulties rather than specific aspects such as frequency or duration, and may therefore partly capture broader psychological distress. Furthermore, the temporal mismatch between the measurement of working from home (past month) and general health (past 12 months) limits the ability to interpret the associations as contemporaneous and raises the possibility of reverse causation. Third, working from home was measured as the number of days worked from home during a typical week, which relies on self-reports over the past month and may therefore be subject to some recall bias. This measure does not capture potential differences in how remote work is organized or experienced. For example, the measure does not distinguish between voluntary and mandatory remote work, differences in home working conditions, or the level of organizational support. Finally, the study did not include detailed information about occupational categories or job types. Because opportunities and requirements for remote work vary substantially across occupations, and work characteristics differ across professional groups, some of the observed associations may partly reflect underlying occupational differences rather than working-from-home arrangements per se. Future research could further examine how the consequences of working from home differ across occupational contexts.

## 5. Conclusions

The present study examined how different degrees of working from home relate to employees’ perceptions of the work environment, job satisfaction, and health. The findings indicate that the outcomes associated with working from home are not uniform but may vary across different working-from-home arrangements. Hybrid work and predominantly home-based work were associated with different patterns of outcomes, suggesting that treating working from home as a single, homogeneous condition may obscure important differences in how work is experienced. More specifically, the findings suggest that working from home may involve shifts in the composition of job demands and resources, rather than uniform effects on the overall level of demands. This may help explain why different aspects of employees’ work experiences are affected in different ways.

More broadly, the results suggest that working from home may be associated with differences in everyday coordination processes within organizations, as reflected in higher levels of role ambiguity. This pattern suggests that changes in where work is performed may be related to how expectations, responsibilities, and interactions are organized in everyday work, and points to the importance of coordination processes as a key dimension of remote work. Taken together, the results suggest that the implications of working from home depend not only on whether employees work remotely but also on how remote work is organized across different working-from-home arrangements, and that coordination-related aspects of work may represent a central but often overlooked dimension in understanding these differences. Furthermore, the findings underscore the importance of considering both different working-from-home arrangements and the multiple dimensions of employee outcomes when organizations design and implement remote work practices.

## Figures and Tables

**Table 1:** Means, standard deviations, and correlations among study variables.

Variable	*M*	*SD*	1.	2.	3.	4.	5.	6.	7.	8.
1. Health	3.69	0.98	–							
2. Sleep	0.73	0.61	−0.42	–						
3. JS	5.47	1.11	0.34	−0.25	–					
4. RA	2.61	1.07	−0.28	0.22	−0.58	–				
5. WL	4.10	1.17	−0.24	0.23	−0.10	0.24	–			
6. Job control	4.44	1.21	0.32	−0.26	0.51	−0.40	−0.39	–		
7. Age	48.22	10.82	0.10	0.07	0.16	−0.12	−0.06 **	0.12	–	
8. Income	13.04	0.54	0.14	−0.04 ^ns^	0.12	−0.04 *	0.13	0.14	0.35	–
9. PD	0.62	0.49	−0.54	0.46	−0.50	0.42	0.29	−0.42	−0.22	−0.12

Health = general health, Sleep = sleep problems, JS = job satisfaction, RA = role ambiguity, WL = workload, Income is log-transformed and winsorized annual income (*M* = 13.04, corresponding to approximately 460 thousand Swedish kronor [SEK]), PD = psychological distress. All correlations statistically significant at *p* < 0.001 except where indicated: * *p* < 0.05. ** *p* < 0.01. ns = not significant.

**Table 2:** Demographic and job-related characteristics by working from home group.

Variable	Work from Home			Test (*F*/χ^2^)
	Never	Hybrid	Majority	
**Demographic Characteristics**				
Age—*M* (*SD*)	49.29 ^a^ (10.83)	47.51 ^b^ (10.90)	47.56 ^b^ (10.37)	7.41 ***
*Sex*				2.35
– Woman—*n* (%)	516 (40%)	540 (42%)	223 (17%)	
– Man—*n* (%)	390 (37%)	462 (44%)	195 (19%)	
*Education*				66.37 ***
– Compulsory—*n* (%)	155 ^a^ (58%)	77 ^c^ (29%)	35 ^b^ (13%)	
– Upper secondary—*n* (%)	317 ^b^ (43%)	296 ^b^ (40%)	128 (17%)	
– University degree—*n* (%)	433 ^c^ (33%)	629 ^a^ (48%)	255 ^a^ (19%)	
Income—*M* (*SD*)	12.93 ^b^ (0.53)	13.09 ^a^ (0.54)	13.15 ^a^ (0.48)	31.78 ***
*Children under 6 years of age*				16.36 ***
– Yes—*n* (%)	93 ^b^ (29%)	164 ^a^ (51%)	66 (20%)	
– No—*n* (%)	813 ^a^ (41%)	838 ^b^ (42%)	352 (17%)	
**Job-related characteristics**				
*Sector*				15.78 ***
– Public sector—*n* (%)	454 ^a^ (42%)	463 (43%)	161 ^b^ (15%)	
– Private sector—*n* (%)	415 ^b^ (36%)	496 (43%)	239 ^a^ (21%)	
*Managerial position*				10.38 **
– Manager—*n* (%)	173 (36%)	237 ^a^ (49%)	71 (15%)	
– Not manager—*n* (%)	732 (40%)	763 ^b^ (41%)	347 (19%)	
Tenure—*M* (*SD*)	12.04 ^a^ (10.22)	9.67 ^b^ (9.24)	9.55 ^b^ (8.22)	15.66 ***
Job control—*M* (*SD*)	4.28 ^b^ (1.25)	4.55 ^a^ (1.15)	4.54 ^a^ (1.22)	13.61 ***

Income is log-transformed and winsorized annual income (group means correspond approximately to 412, 484, and 514 thousand SEK). *F*-tests for continuous variables and χ^2^-tests for categorical variables. For continuous variables, different superscript letters indicate significant differences between WFH categories. For categorical variables, superscripts indicate statistically significant differences between categories within each WFH category (Bonferroni-adjusted *p* < 0.017). ** *p* < 0.01. *** *p* < 0.001.

**Table 3:** Multivariate regression models predicting work environment, job attitudes, and health outcomes.

Variable	Health	Sleep	Job Satisfaction	Role Ambiguity	Workload
Hybrid Work	−0.03 (0.04)	0.01 (0.03)	−0.15 (0.05) **	0.26 (0.05) ***	−0.01 (0.05)
Majority WFH	−0.21 (0.06) ***	0.10 (0.04) **	−0.24 (0.06) ***	0.31 (0.06) ***	−0.18 (0.06) **
*Control variables*					
Position	−0.03 (0.05)	0.00 (0.03)	0.20 (0.05) ***	−0.07 (0.05)	0.60 (0.06) ***
Sex (woman)	−0.05 (0.04)	0.04 (0.03)	0.22 (0.04) ***	−0.16 (0.04) ***	0.04 (0.05)
Private sector ^a^	0.11 (0.04) **	−0.01 (0.03)	−0.17 (0.04) ***	0.06 (0.04)	−0.06 (0.05)
Upper secondary ^b^	−0.01 (0.07)	0.01 (0.04)	−0.08 (0.07)	0.01 (0.07)	0.03 (0.08)
University degree ^b^	0.08 (0.07)	−0.03 (0.04)	−0.11 (0.07)	0.07 (0.07)	0.15 (0.08)
Job control	0.24 (0.02) ***	−0.13 (0.01) ***	0.46 (0.02) ***	−0.37 (0.02) ***	−0.42 (0.02) ***
Age	0.00 (0.00)	0.01 (0.00) ***	0.01 (0.00) *	−0.01 (0.00) **	−0.01 (0.00) ***
Income	0.17 (0.05) ***	−0.04 (0.03)	0.13 (0.05) **	0.02 (0.05)	0.41 (0.05) ***
*R* ^2^	0.13	0.09	0.29	0.19	0.25

Position = managerial position. WFH = working from home. Never working from home is the reference group. Unstandardized coefficients with standard errors in parentheses. Income is log-transformed and winsorized annual income. ^a^ Reference category: Public sector. ^b^ Reference category: Compulsory school. * *p* < 0.05. ** *p* < 0.01. *** *p* < 0.001.

## Data Availability

The raw data supporting the conclusions of this article will be made available by the author upon reasonable request.

## Supplementary Materials

The following supporting information can be downloaded at: https://www.mdpi.com/article/10.3390/ijerph23040524/s1, Table S1: Demographic and job-related characteristics by working from home group. Table S2: Multivariate regression model predicting work environment, job attitudes, and health outcomes including psychological distress as an additional control variable.

## Abbreviations

The following abbreviation is used in this manuscript:

WFH	Working from home
