# Supplementary material for: Working from Home: Hybrid and Predominantly Home-Based Work in Relation to Work Environment, Job Satisfaction, and Health
Source: Int J Environ Res Public Health. 2026 Apr 18;23(4):524. doi: 10.3390/ijerph23040524 (PMC13116910; doi:10.3390/ijerph23040524)
Supplement: Supplementary file 1 [file ijerph-23-00524-s001.zip › ijerph-4227405-supplementary.pdf]

Supplementary Materials

To assess the robustness and generalizability of the descriptive findings, all comparisons reported in Table 2 were re-estimated using survey-weighted analyses based on calibrated population weights provided by Statistics Sweden. These weights adjust for non-response and align the sample with the Swedish workforce with respect to key demographic and labor market characteristics derived from population register data. The results are presented in Table S1.

Table S1. Demographic and job-related characteristics by working from home group.

Variable	Work from Home			Test (F)
	Never	Hybrid	Majority	
**Demographic Characteristics**				
Age — M (SE)	45.83 (.42)	43.89 (.37)	44.01 (.54)	6.67**
Sex				3.37*
– Woman —% (SE)	43% (.02)	40% (.02)	17% (.01)	
– Man — % (SE)	37% (.02)	44% (.02)	19% (.01)	
Education				22.49***
– Compulsory — % (SE)	65% (.03)	24% (.03)	11% (.02)	
– Upper secondary — % (SE)	46% (.02)	37% (.02)	17% (.02)	
– University degree — % (SE)	32% (.01)	48% (.02)	20% (.01)	
Income — M (SE)	12.83 (.02)	12.98 (.02)	13.07 (.03)	20.91***
Children under 6 years of age				6.64**
– Yes — % (SE)	31% (.03)	48% (.03)	21% (.02)	
– No — % (SE)	42% (.01)	40% (.01)	18% (.01)	
**Job-related characteristics**				
Sector				6.66**
– Public sector — % (SE)	43% (.02)	42% (.01)	15% (.01)	
– Private sector — % (SE)	37% (.02)	42% (.02)	21% (.01)	
Managerial position				3.81*
– Manager — % (SE)	36% (.02)	48% (.03)	15% (.02)	
– Not manager — % (SE)	40% (.01)	41% (.01)	19% (.01)	
Tenure — M (SE)	10.65 (.35)	8.53 (.29)	8.08 (.38)	15.24***
Job control — M (SE)	4.20 (.05)	4.54 (.04)	4.46 (.07)	15.38***

Income is log-transformed and winsorized annual income. Values for continuous variables are survey-weighted means with standard errors in parentheses. Categorical variables are presented as row proportions with linearized standard errors. All analyses are based on calibrated population weights provided by Statistics Sweden. *$p < .05$. **$p < .01$. ***$p < .001$.

The results closely mirror those obtained in the unweighted analyses. The overall pattern of differences across working-from-home categories remains unchanged, indicating that the findings are robust to the use of calibrated survey weights and supporting the generalizability of the findings.

In addition, as a sensitivity analysis, the multivariate regression model was re-estimated including psychological distress as an additional control variable (see Table S2). The overall pattern of results remained unchanged after this adjustment, even though this variable was strongly associated with all outcomes. This suggests that the observed associations between workingfrom-home arrangements and the study outcomes are not simply attributable to underlying differences in psychological health. Joint multivariate tests indicated that working-from-home status was significantly associated with the set of outcomes, $F(10, 2197) = 7.24$, $p < 0.001$. Both hybrid work (≤2 days) and predominantly home-based work (majority WFH, 3–5 days) showed significant associations across outcomes, $F(5, 2197) = 7.19$, $p < 0.001$, and $F(5, 2197) = 10.39$, $p < 0.001$, respectively. The coefficients also differed significantly between hybrid and predominantly home-based work, $F(5, 2197) = 4.64$, $p < 0.001$.

Table S2. Multivariate regression model predicting work environment, job attitudes, and health outcomes including psychological distress as an additional control variable.

Variable Health Sleep	Variable Health Sleep	Variable Health Sleep	Variable Health Sleep	Variable Health Sleep	Variable Health Sleep
Hybrid Work	−0.02 (0.04)	0.01 (0.02)	−0.15 (0.04) ***	0.26 (0.04) ***	−0.02 (0.05)
Majority WFH	−0.16 (0.05) ***	0.07 (0.03) *	−0.21 (0.06) ***	0.28 (0.06) ***	−0.20 (0.06) ***
Control variables					
Managerial position	−0.07 (0.04)	0.02 (0.03)	0.18 (0.05) ***	−0.05 (0.05)	0.62 (0.06) ***
Sex (woman)	−0.06 (0.04)	0.05 (0.02) *	0.21 (0.04) ***	−0.15 (0.04) ***	0.04 (0.05)
Private sector [a]	0.10 (0.04) **	0.00 (0.02)	−0.17 (0.04) ***	0.07 (0.04)	−0.05 (0.04)
Upper secondary [b]	0.04 (0.06)	0.02 (0.04)	−0.04 (0.07)	−0.02 (0.07)	0.01 (0.08)
University degree [b]	0.11 (0.06)	−0.05 (0.04)	−0.08 (0.07)	0.04 (0.07)	0.13 (0.08)
Job control	0.09 (0.02) ***	−0.04 (0.01) ***	0.34 (0.02) ***	−0.26 (0.02) ***	−0.35 (0.02) ***
Age	−0.00 (0.00)	0.01 (0.00) ***	0.00 (0.00)	−0.00 (0.00)	−0.01 (0.00) **
Income	0.16 (0.04) ***	−0.04 (0.03)	0.12 (0.04) **	0.03 (0.05)	0.41 (0.05) ***
PD	−0.97 (0.04) ***	0.57 (0.02) ***	−0.75 (0.04) ***	0.64 (0.04) ***	0.41 (0.05) ***
R^2	0.32	0.26	0.37	0.26	0.27

Never working from home is the reference group. Unstandardized coefficients with standard errors in parentheses. Income is log-transformed and winsorized annual income. PD = psychological distress. aReference category: Public sector. bReference category: Compulsory school. N = 2209. * $p < 0.05$. ** $p < 0.01$. *** $p < 0.001$.
